# Race disparities in total PM_2.5_ and wildfire-specific PM_2.5_ exposure among disaggregated Asian American, Native Hawaiian, and Pacific Islander groups in California

**DOI:** 10.1016/j.joclim.2026.100755

**Published:** 2026-10-29

**Authors:** Minhazul Mohsin, Mengyi Li, Jun Wu, Sora Park Tanjasiri, Brittany N. Morey

**Affiliations:** a Department of Health, Society, & Behavior, Joe C. Wen School of Population & Public Health, University of California, Irvine, USA; b Department of Environmental & Occupational Health, Joe C. Wen School of Population & Public Health, University of California, Irvine, USA; c Chao Family Comprehensive Cancer Center, University of California, Irvine, USA

**Keywords:** PM_2.5_, Wildfire-specific PM_2.5_, Racial disparities, Asian Americans, Native Hawaiians and Pacific Islanders, Environmental justice

## Abstract

**Introduction:**

Environmental health disparities in the United States (US) are evident in PM_2.5_ exposure, but few studies have documented wildfire-specific PM_2.5_ disparities. Furthermore, aggregating broad racial groups may hide disparities for Asian American (AA) and Native Hawaiian or Pacific Islander (NHPI) groups. We investigated racial disparities in total and wildfire-specific PM_2.5_ exposure in California and determined whether disaggregating AA and NHPI groups revealed previously hidden disparities.

**Methods:**

We conducted a cross-sectional descriptive analysis using data from the US Environmental Protection Agency’s Air Quality System data (2008-2012) to assess average total PM_2.5_ levels in census tracts, including wildfire-specific PM_2.5_. The 2010 U.S. Census provided census tract level counts for American Indian/Alaska Native, AA, Black, NHPI, White, and 19 disaggregated AA and 10 disaggregated NHPI groups. We calculated population-weighted average exposure to total PM_2.5_ and wildfire-specific PM_2.5_ for all race groups across California state.

**Results:**

Results showed differences in the distribution of total PM_2.5_ compared to wildfire-specific PM_2.5_ in the state. Disaggregated data revealed disparities, including high exposure to total PM_2.5_ among several disaggregated AA and NHPI groups and Black populations. Hmong populations experienced the highest average exposure to total PM_2.5_ and wildfire-specific PM_2.5_ compared to all other race groups. The highest average wildfire-specific PM_2.5_ exposure was observed among Hmong, Fijian, Laotian, Bhutanese, Marshallese, and other Micronesian populations.

**Conclusion:**

Race disparities exist for total and wildfire-specific PM_2.5_. Disaggregating AA and NHPI groups reveals disparities among certain populations. This study informs future community-based efforts to assess and address health risks associated with exposure to PM_2.5_.

## 1 Introduction

Exposure to PM_2.5_ (particulate matter with an aerodynamic diameter <2.5 micrometers) is the main contributor to air pollutant-caused disease [1], and is associated with 85,000–200,000 excess deaths per year in the United States (US) [2,3]. PM_2.5_ is harmful as it penetrates deep into the lung and may enter into other vital organs through the bloodstream [4]. Higher PM_2.5_ exposure is linked to a range of respiratory and cardiovascular diseases, as well as several cancers, including lung, breast, colorectal, and prostate cancer [[5], [6], [7], [8], [9]]. Although PM_2.5_ concentrations have decreased by 37% from 2000 to 2023 [10], racial and ethnic disparities in air pollution exposure persist in the US [2,11]. African American/Black (henceforth referred to as Black), American Indian/Alaska Native (AIAN), Asian, and Hispanic populations are exposed to higher PM_2.5_ compared to White populations [2,12].

California has among the highest PM_2.5_ concentrations in the United States, and despite decades of active vehicle emission policies, racial and ethnic disparities in PM_2.5_ exposure have persisted or widened [13]. Although existing data show persistent disparities in PM_2.5_ exposure, there have been few studies examining race disparities in wildfire-specific PM_2.5_ exposure. Wildfire incidence is increasing in the US partially due to climate change [14,15]. While overall PM_2.5_ is on the decline, wildfires cause significantly elevated PM_2.5_ emissions in the affected regions [16]. Wildfire-specific PM_2.5_ may have unique toxicological effects compared to PM_2.5_ from non-fire sources [17] and has been linked to higher disease risk [18]. However, it is unknown how race disparities differ for total PM_2.5_ compared to wildfire-specific PM_2.5_. Research indicates that Black populations experience higher exposure to wildfire smoke compared to White populations [19,20]. Additionally, a recent study found that AIAN, and multiracial communities are consistently over-represented among those exposed to wildfire-specific PM_2.5_ [21]. More research is needed to identify other communities of color may be at risk of wildfire-specific PM_2.5_ exposure given the increasing threat of climate change.

To illuminate race disparities in PM_2.5_ exposure, it is important not only to examine the minimum race groups as defined by the Office of Management and Budget (OMB) between 1997 and 2024 – AIAN, Asian American (AA), Black, Native Hawaiian/Pacific Islander (NHPI), and White – but also to disaggregated subgroups, especially for AAs and NHPIs. The aggregate AA and NHPI categories fail to account for the diversity within these categories [[22], [23], [24]], which encompass unique countries of origin, languages, cultural practices, and histories of migration in the US. AAs include specific groups such as Bangladeshi, Cambodian, Chinese, Hmong, Indian, Japanese, Korean, Vietnamese, and many others. The US categorizes NHPIs into three major groupings of islands in the Pacific Ocean: Polynesians (including Native Hawaiian, Samoan, and Tongan), Micronesians (including Chamorro/Guamanian and Marshallese), and Melanesians (including Fijian) [25]. However, most studies examining environmental justice issues leave out AA and NHPI populations, or erroneously aggregate AAs with NHPIs, obscuring race disparities. Furthermore, no study to date has examined PM_2.5_ exposure comprehensively among disaggregated AA and NHPI groups. Health studies show subgroup variation in disease risk [26,27]. For instance, using pooled national cancer registry data, one study reported that lung cancer was most common in Chamorro, Micronesians, and Vietnamese populations whereas colorectal cancer was most common in Cambodian, Laotian, and Papua New Guinean populations [28]. Disease disparities suggest that the underlying risk factors, including PM_2.5_ exposure, may also vary among subgroups [29,30].

This study investigates differences in population weighted average exposure to total and wildfire-specific PM_2.5_ across disaggregated AA and NHPI groups, as well as the 5 minimum OMB race groups ( AA, AIAN, Black, NHPI, and White), in California using data from the U.S. Census. We focus our analysis on California, a state that is one of the most impacted by wildfires and consistently ranks among the highest in wildfire risk [31]. California is additionally the state with the largest population of AAs and the second largest population of NHPIs (after Hawaii) in the US [25]. The objective of this study was to quantify and compare total and wildfire-specific PM_2.5_ exposure among disaggregated AA and NHPI subgroups and to examine disparities relative to aggregated AA, AIAN, Black, NHPI, and White populations in California.

## 2 Material and methods

### 2.1 Data sources

We conducted a cross-sectional study utilizing data from the US Environmental Protection Agency (EPA) Air Quality System (2008-2012) and the 2010 US Census. We used 2010 Census data to obtain population counts by race groups for all 2010 census tracts across the state of California. No minimum population or census tract threshold was applied; all AA and NHPI population groups tabulated by the 2010 Census for California were reported.

In 2010, the OMB’s Statistical Policy Directive No. 15, minimum standard of data collection for race, included AIAN, AA, Black, NHPI, and White. In addition, we included data for 19 specific AA groups (Bangladeshi, Bhutanese, Burmese, Cambodian, Chinese, Filipino, Hmong, Indian, Indonesian, Japanese, Korean, Laotian, Malaysian, Nepalese, Pakistani, Srilankan, Taiwanese, Thai, Vietnamese) and 10 specific NHPI groups (Chamorro/Guamanian, Fijian, Marshallese, Native Hawaiian, Samoan, Tongan, other Polynesian [not including Native Hawaiian, Samoan, or Tongan], other Micronesian [not including Chamorro/Guamanian or Marshallese], other Melanesian [not including Fijian], and other Pacific Islander not otherwise specified) from 2010 Census data for all of California. AA and NHPI race groups were not mutually exclusive; each AA and NHPI group included those who identified as that group alone, or in combination with any other race or ethnic group. This approach was due to many NHPI individuals identifying as multiracial, and prior research showing a strong preference for multiracial NHPIs to be counted as NHPI [32,33]. The 2010 Census collected Hispanic or Latino origin as ethnicity, separately from race, and allowed individuals of Hispanic or Latino origin to identify with any race [34]. This analysis did not include a separate Hispanic or Latino group; rather, we focused on the OMB definitions of race in 2010, under which people of any of the included race groups could be of any ethnicity (i.e., Hispanic or non-Hispanic).

We used the 2010 Census population counts for disaggregated AA and NHPI groups to minimize measurement error in minoritized populations. Evidence from the 2020 Census reveals substantial response shortfalls and patterns consistent with differential undercounts in disadvantaged and racially minoritized neighborhoods in Los Angeles County, and discrepancies with the American Community Survey, which could introduce additional uncertainty for small-area subgroup denominators [35,36]. As our main aim is to quantify disparities among disaggregated AAs and NHPIs, we chose the 2010 Census, which has the strongest reliability for subgroup population counts.

Data on exposure to total PM_2.5_ and wildfire-specific PM_2.5_ came from a machine-learning model that was built using PM_2.5_ monitoring data from the US EPA Air Quality System (2006-2020), with explanatory variables such as satellite-based aerosol properties, meteorological conditions, land-use data, thermal inversions, and wildfire-related data including smoke plume data from the National Oceanic and Atmospheric Administration (NOAA) Hazard Mapping System and wildfire perimeter polygons from the Fire and Resource Assessment Program [37]. In brief, to estimate daily PM_2.5_ levels at a fine resolution, machine learning models (including deep learning, distributed random forest, and gradient boosting) were trained and combined using ensemble learning. To isolate wildfire-specific PM_2.5_, a multiple imputation approach was used to estimate non-smoke PM_2.5_ concentrations, which were then subtracted from the total PM_2.5_ levels. Estimated PM_2.5_ concentrations were validated against existing datasets and the smoke density classifications from the NOAA Hazard Mapping System. Further details on the modeling, validation, and data sources can be found in the main modeling paper [37].

### 2.2 Data analysis

Air pollution levels were analyzed at the 2010 census tract level across California state. Daily total PM_2.5_ and wildfire-specific PM_2.5_ estimates were obtained from the published machine-learning model described above. For each census tract i, we computed the annual mean concentration for each year of 2008-2012 and then calculated the 5-year mean concentration at the census tract level $C_{ip}$ (where p denotes total PM_2.5_ or wildfire-specific PM_2.5_). We then computed the population-weighted mean exposure *E_gp_* for each race group g using census tract-level race-specific population counts $Pop_{ig}$ by summing the multiples of the census tract-level PM_2.5_ exposures and race-specific population counts, then dividing the sum by the total race-specific population counts across all of California. This is represented by the equation:

$$\bar{E_{gp}}=\frac{\sum_{i=1}^{N}Pop_{ig}\;*C_{ip}}{\sum_{i=1}^{N}Pop_{ig}}$$

This yields a statewide, population-weighted mean exposure for each race group during 2008-2012, rather than a sample-based estimate. We selected 5-year annual average PM_2.5_ in census tracts (2008–2012) to align the air pollution data with the 2010 Census populations, ensuring demographic relevance and consistency with census data. This time frame also provides a stable baseline of total PM_2.5_ and wildfire-specific PM_2.5_ exposure, capturing both typical air quality patterns and moderate wildfire season without being skewed by extreme wildfire events like in 2017, 2018 and 2020.

We visualized the 5-year annual average total and wildfire-specific PM_2.5_ concentrations across California Census tracts using package “ggplot2” in R (version 4.3.0). The resulting population-weighted mean exposure estimates for each race group were plotted as bar graphs for total and wildfire-specific PM_2.5_, ordered from highest to lowest, to compare exposure across groups. We plotted AA specific groups separately from NHPI specific groups; estimates for the 5 minimum OMB race groups (AIAN, AA, Black, NHPI, and White) were displayed in each bar graph. The presented comparisons are descriptive, population-weighted contrasts. Our estimates are derived from complete 2010 census data rather than a probability sample. Therefore, the population-weighted means represent observed population parameters rather than sample-based estimates subject to sampling error. This applies to all subgroups including the smallest disaggregated subgroups. Each subgroup mean is calculated from a complete count of that subgroup, so minimum sample size thresholds do not apply. Frequentist hypothesis testing is therefore not applicable in this context.

## 3 Results

### 3.1 Total PM_2.5_ and wildfire-specific PM_2.5_ in California census tracts

Fig. 1 shows maps of 2008-2012 5-year average total and wildfire-specific PM_2.5_ in California census tracts. Darker purple areas show higher average levels of PM_2.5_, while yellow areas show lower average levels of PM_2.5_. The statewide 5-year population-weighted mean exposure to total PM_2.5_ was 11.29 µg/m³, and wildfire-specific PM_2.5_ was 0.38 µg/m³. Average total PM_2.5_ varied considerably across the state. In Fig. 1a, average total PM_2.5_ appears to be highest in the Central Valley, which is bounded by mountain ranges and is known for agriculture and meteorology conducive to fine particulate matter formation [38]. Fig. 1b shows average wildfire-specific PM_2.5_ was highest in northernmost California. The extensive forested areas and frequent wildfires in Northern California likely contributed to the higher levels, aligning with previously reported spatial patterns of wildfire-specific PM_2.5_ across California [20].

**Fig. 1:**
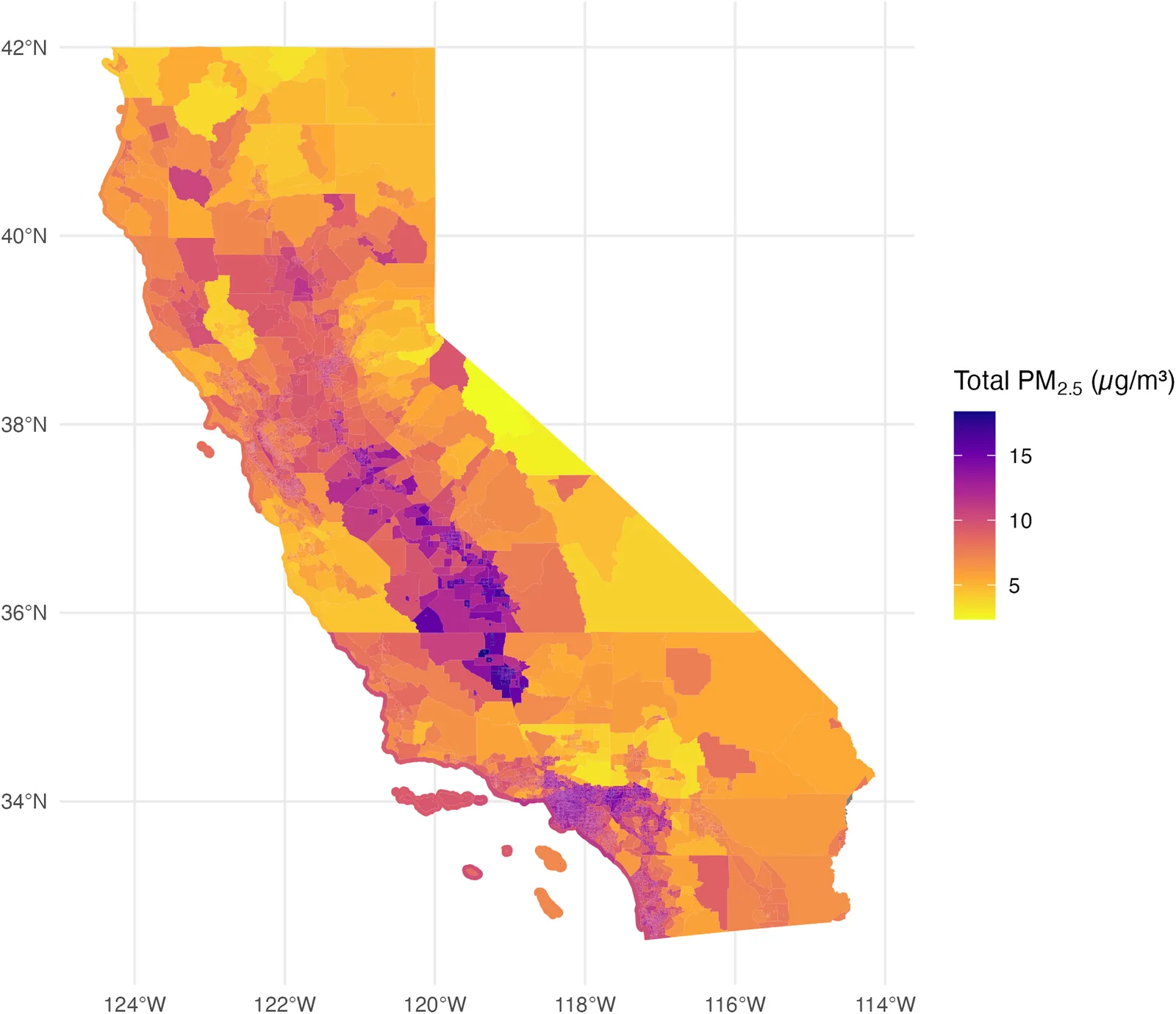

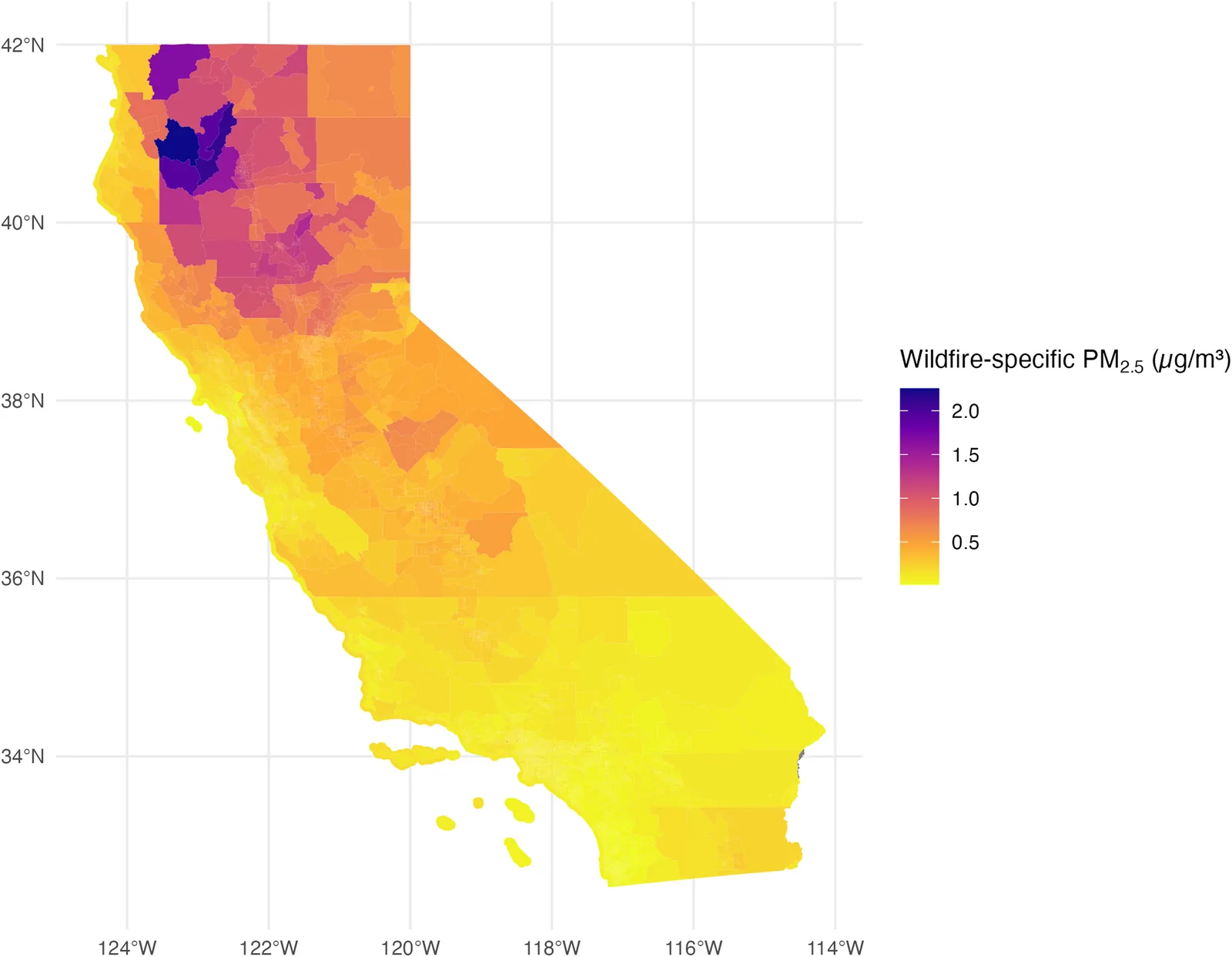
Annual average total PM_2.5_ and wildfire-specific PM_2.5_ in California Census tracts (2008-2012).

1a. Annual average total PM_2.5_ in California; 1b. Annual average wildfire-specific PM_2.5_ in California; Note: Data from the US Environmental Protection Agency Air Quality System.

### 3.2 Asian American exposure to total PM_2.5_ and wildfire-specific PM_2.5_ compared to others

Fig. 2 compares population-weighted average exposures to total and wildfire-specific PM_2.5_ by race. The red vertical line indicates the US EPA's maximum annual standard PM_2.5_ exposure, set at 9 µg/m³ in 2024 [10]. In California, all racial groups, including the disaggregated AA and NHPI groups, experienced PM_2.5_ levels above this threshold.

**Fig. 2:**
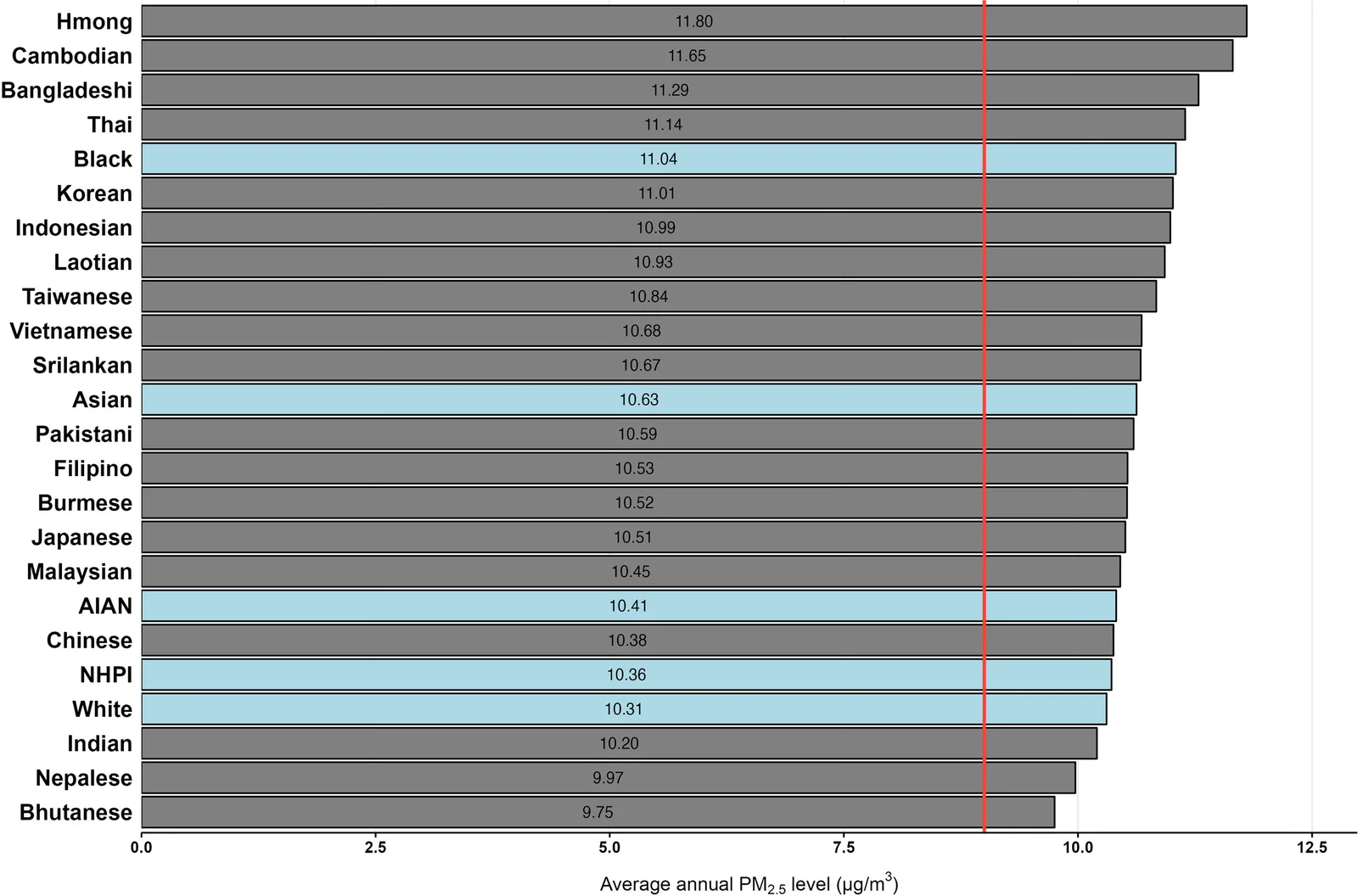

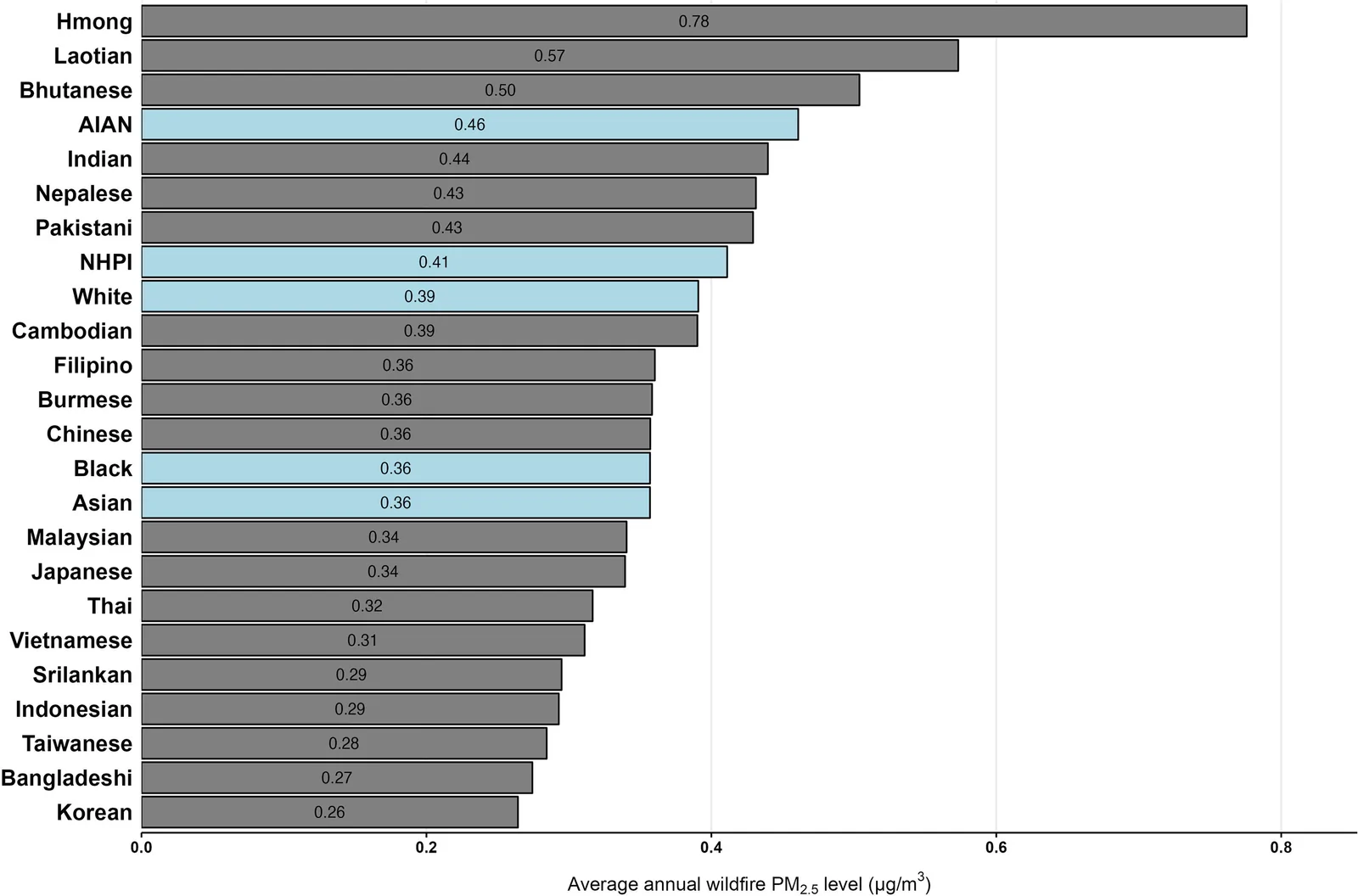

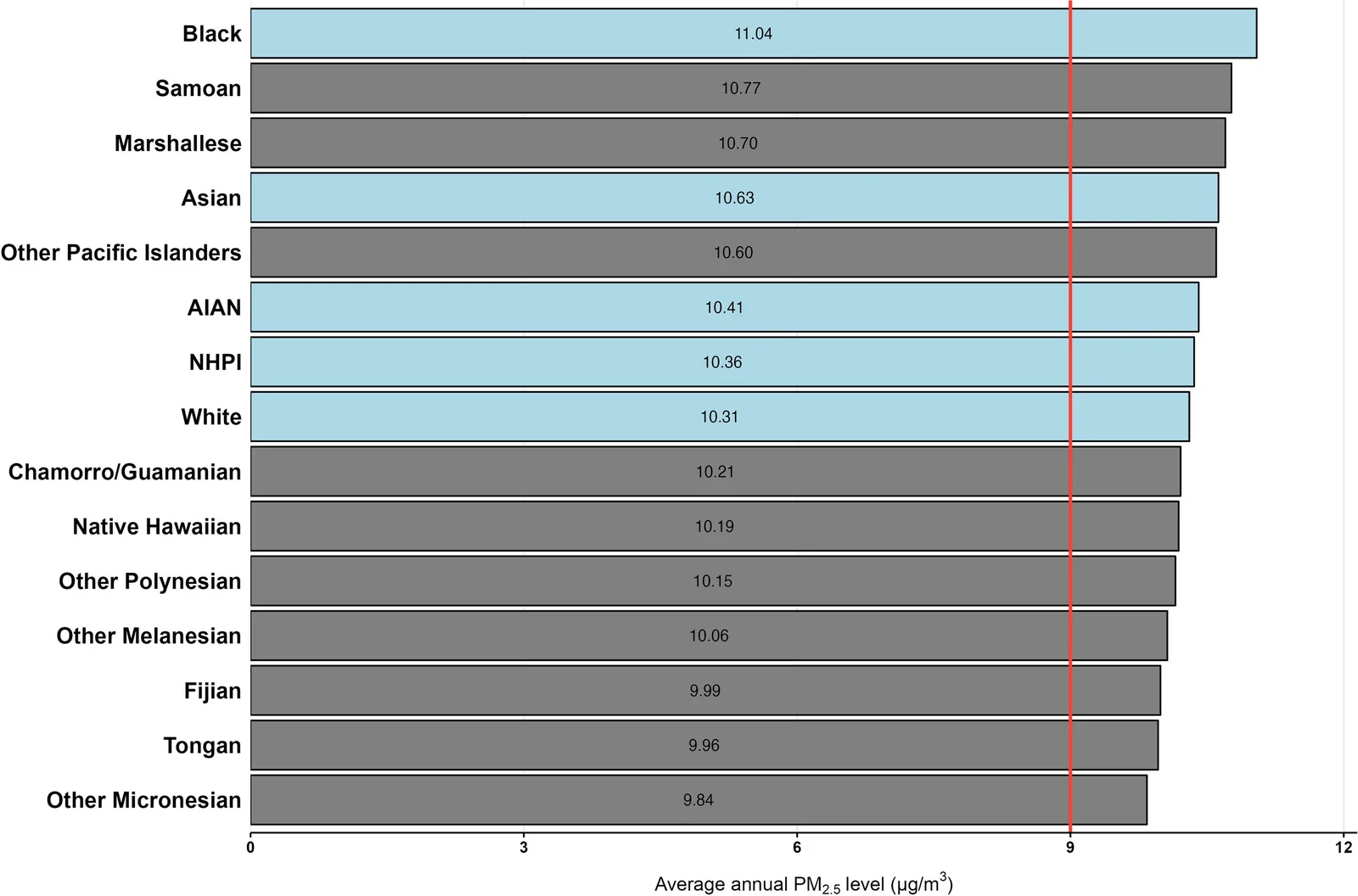

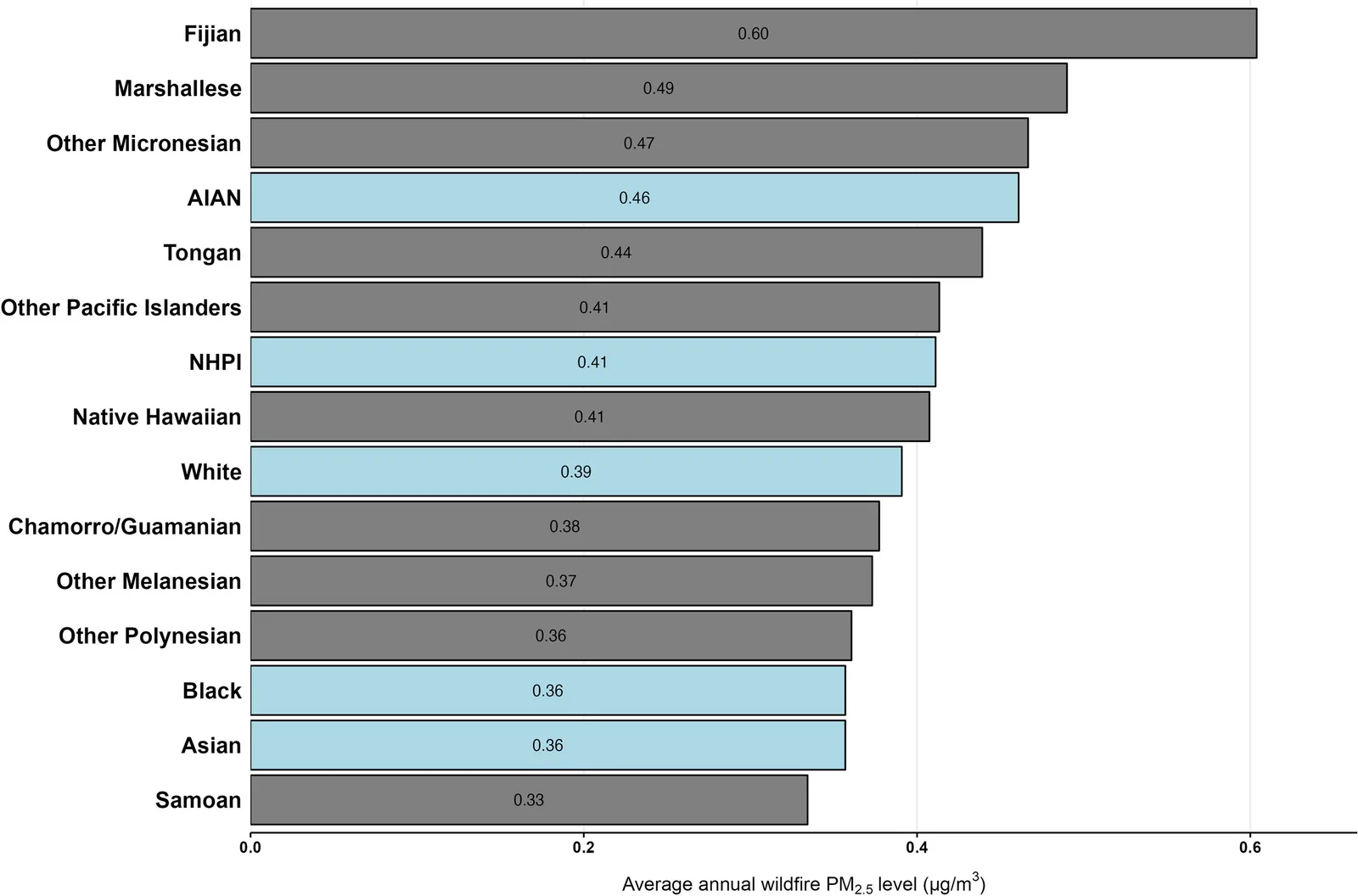
Population-weighted annual average exposure to total PM_2.5_ and wildfire-specific PM_2.5_ by disaggregated race group (2008-2012).

2a. Average total PM_2.5_ exposure by disaggregated AA race; 2b. Average wildfire-specific PM_2.5_ exposure by disaggregated AA race; 2c. Average total PM_2.5_ exposure by disaggregated NHPI race; 2d. Average wildfire-specific PM_2.5_ exposure by disaggregated NHPI race; Notes: Data from US Environmental Protection Agency Air Quality System and 2010 US Census. AIAN = American Indian/Alaska Native; AA = Asian American, NHPI = Native Hawaiian or Pacific Islander.

Fig. 2a shows the average total PM_2.5_ exposure for AA-specific groups and five OMB race groups. Hmong had the highest exposure at 11.80 µg/m³, followed by Cambodians, Bangladeshis, and Thais. Blacks had the next highest exposure at 11.04 µg/m³, comparable to Koreans and Indonesians. Compared to Whites, Hmong were exposed to 14.45% higher PM_2.5_ while Cambodians were 13% higher. The next groups were Laotians, Taiwanese, Vietnamese, and Sri Lankans, who all faced higher exposure than the aggregated AA at 10.63 µg/m³. Pakistanis, Filipinos, Burmese, Japanese, and Malaysians all had lower exposure than aggregated AAs, but still higher than AIANs and Whites. Chinese had higher total PM_2.5_ exposure compared to Whites. Asian Indians, Nepalese, and Bhutanese had the lowest average PM_2.5_ exposure of all other race groups and were the only groups with 1–5% lower exposure than that of Whites.

Fig. 2b illustrates wildfire-specific PM_2.5_ exposure across the AA subgroups and five OMB race groups. Hmong had the highest average exposure at 0.78 µg/m³, which was double the exposure faced by Whites, followed by Laotians and Bhutanese who were 46.15% and 28.21% higher than Whites, respectively. AIANs had the next highest, followed by Asian Indians, Nepalese, and Pakistanis. All these groups, including aggregated NHPIs, had higher wildfire-specific PM_2.5_ exposure than Whites. Cambodians had similar exposure to that of Whites (0.39 µg/m³). Filipinos, Burmese, and Chinese all had higher wildfire-specific PM_2.5_ than Blacks and aggregated AAs. Malaysians and Japanese had slightly lower exposure compared to aggregated AAs. Thais, Vietnamese, Sri Lankans, Indonesians, and Taiwanese experienced relatively lower wildfire-specific PM_2.5_ exposure. Bangladeshi and Korean groups had the lowest levels of exposure with Koreans averaging 0.26 µg/m³, which was 33.33% lower than Whites.

### 3.3 Native Hawaiian and Pacific Islander exposure to total PM_2.5_ and wildfire-specific PM_2.5_ compared to others

Fig. 2c shows the average total PM_2.5_ exposure for NHPI specific groups and the five OMB race groups. Among NHPIs, Samoans and Marshallese had the highest exposure – 4.46% and 3.78% higher exposure than Whites. Blacks had the highest exposure, just above the Samoans. Aggregated AAs followed Marshallese, with a similar level of exposure as the other Pacific Islander race group. AIANs (10.41 µg/m³) and NHPIs in aggregate (10.36 µg/m³) had slightly higher average (about 0.48%) PM_2.5_ exposure as Whites (10.31 µg/m³). Chamorro/Guamanians, Native Hawaiians, Other Polynesians, Other Melanesians, Fijians, Tongans, and Other Micronesians all had relatively lower exposure to total PM_2.5_ than Whites, although this was still higher than the EPA recommended annual standard level of 9 µg/m³.

Fig. 2d shows wildfire-specific PM_2.5_ exposure among NHPI and the five OMB race groups. Fijians had the highest average exposure at 0.60 µg/m³, about 53.85% higher than Whites. This was followed by Marshallese and other Micronesians, who had average exposure levels about 25.64% and 20.51% higher than Whites. These average exposures were higher than aggregated AIANs. Tongans and Other Pacific Islanders (not otherwise specified) also had higher exposure than aggregated NHPIs. Native Hawaiians had similar exposure as aggregated NHPIs, which was higher than the average exposure for Whites. The average wildfire-specific PM_2.5_ exposure for the aggregated NHPI was 0.41 µg/m³ which was about 5.12% higher than Whites. Chamorro/Guamanians, Other Melanesians, and Other Polynesians had moderate exposure, just below Whites and above Blacks and AAs. Samoans had the lowest average exposure compared to other NHPI groups, at 0.33 µg/m³, about 15.38% lower than that of Whites.

## 4 Discussion

We examined disparities in exposure to total and wildfire-specific PM_2.5_ across the OMB race groups and disaggregated AA and NHPI groups in California. Overall, AA and NHPI populations had among the highest burdens of total and wildfire-specific PM_2.5_ exposure, with pronounced disparities in disaggregated groups. AA groups including Hmong, Cambodians, and Thais faced higher average total PM_2.5_ exposure while Hmong and Laotians were among the most affected by wildfire-specific PM_2.5_. Among NHPI disaggregated groups, Samoans and Marshallese were exposed to higher total PM_2.5_, while Fijians and Marshallese faced higher wildfire-specific PM_2.5_. These disparities were only evident through disaggregated data, as these levels of exposure were markedly higher than the aggregated AA and NHPI groups. Our findings highlight the importance of disaggregating data to reveal significant environmental injustices which were previously obscured due to data aggregation [21,22,39,40]. This exposure assessment is grounded in environmental epidemiology, where understanding historical exposure windows is essential because pollution-related health effects accumulate over long latency periods [41]. While much of the climate health literature focuses on short-term or acute effects, the long-term cumulative impacts of repeated exposure remain understudied. Estimating chronic historical exposures is therefore necessary to characterize life-course risk and population burden.

Our findings underscore persistent disparities in air pollution exposure, even amid a general decline in PM_2.5_ levels over the last two decades. Previous studies have investigated racial and ethnic disparities in air pollution exposure with a primary focus on minimum OMB race groups and found AIAN, Asian, Black, and NHPI populations faced higher PM_2.5_ exposure than White populations [2,42,43]. While we found similar disparities for total PM_2.5_, our study expands on this literature by examining both total and wildfire-specific PM_2.5_ in California, a state particularly vulnerable to climate change-driven wildfires. To our knowledge, no other studies have examined race disparities in average wildfire-specific PM_2.5_ exposure with this level of granularity. Our findings suggest that there is a differential risk associated with PM_2.5_ exposure due to the unequal spatial distribution of racial groups, with communities experiencing varying levels of risk depending on the pollution sources. Although the long-term average of wildfire-specific PM_2.5_ in this study appeared low due to five-year averaging that predominantly included non-fire days, the relative differences in these averages compared to White and aggregated AA and NHPI groups revealed stark disparities. We further recognize that wildfire exposure is acute and episodic [44], so that averaging over a long period flattens the impact. This suggests that the disparities that we uncovered for five-year averages of wildfire-specific PM_2.5_ may in fact be even more severe, as the populations that experienced higher annual averages likely experienced acute exposure during the actual wildfire events that could be magnitudes higher than their less-exposed counterparts.

The spatial distribution of populations most impacted by total and wildfire-specific PM_2.5_ is closely linked with historical patterns of residential segregation by race and by socioeconomic status in the state. Examining the spatial distribution of total PM_2.5_ shows that higher average PM_2.5_ concentrations are near primary emission sources such as industrial areas in Los Angeles and downwind industrial zones (e.g., Riverside/San Bernardino) [45] – areas that experienced historical redlining and where many low-income communities of color currently live [46]. On the other hand, areas with high average wildfire-specific PM_2.5_ exposure tend to be rural or wildland-urban interface areas (i.e., places where human development intermingles with undeveloped wildland) [47]. Residents of these areas may live there due to historical exclusion and current economic realities which contribute to persistent racial and ethnic disparities [48,49]. A notable example is the Hmong population, many of whom resettled in rural and agricultural regions of the Central Valley following displacement from their homeland during the 1970s due to persecution and war [50]. Many Hmong people who worked in occupations such as agriculture in their homeland continue to work in sectors such as agriculture and manufacturing in the US. Hmong communities face significant social and economic barriers in the US, including poverty and lack of access to healthcare. In our analysis, Hmong communities experienced the highest average exposures to both total and wildfire-specific PM_2.5_, suggesting how historically displaced and marginalized populations face environmental injustice in the US. These environmental injustices contribute to downstream health inequities, warranting the need for targeted interventions to mitigate and address these disparities [51].

While our findings are unique, there are limitations to be noted. The data are cross-sectional and cannot examine trends over time or migration-related changes in exposure. Wildfire activity and smoke transport patterns in California have shifted over time; therefore, the results of this study should be interpreted as a historical baseline of exposure disparities for the study period, rather than as a prediction of future wildfire conditions. More recent, time-varying maps of wildfire-specific PM_2.5_ across California provide a point of comparison to the historical distribution presented in this study [21]. Whether the disparities we document among disaggregated subgroups have persisted, intensified, or shifted is an important future question that our approach is well suited to address through continued surveillance. Although our study did not aim to uncover current conditions, our findings align with recent evidence indicating that, despite overall improvements in air quality over the past several decades, there are persistent and widening disparities in air pollution-related morbidity and mortality among communities of color [52]. Further, recent evidence indicates that improvements in air quality have begun to slow or reverse from the public health gains achieved over previous decades due to increases in wildfire smoke [53]. In California, specifically, the health burden attributable to wildfire-related PM_2.5_ has offset the public health gains previously achieved through reductions in non-smoke PM_2.5_ [54]. The communities we identified as most exposed including Hmong, Laotian, Fijian, and Marshallese populations are concentrated in wildfire-prone and historically overburdened areas. The worsening air pollution trends reported in recent literature are, therefore, likely to deepen the disparities reported here. Documenting a historical baseline remains valuable because such burdens can persist and intensify in disproportionately burdened communities. This analysis assumes exposure using the proxy of population residence within census tracts. It does not capture how exposure may be affected by other factors such as housing type, time spent outdoors, individual-level vulnerability (e.g., age, disability, chronic disease status, etc.), and time spent at home (versus at work, at school, at place of worship, etc.). Furthermore, because exposure to wildfire smoke is strongly determined by proximity to wildfire-prone regions [55], the disparities observed between groups reflect both spatial variation in PM_2.5_ levels and residential patterns of different groups. These are shaped by historical and structural inequalities, as discussed above. Our analysis quantifies group-level exposure burden but cannot statistically disentangle the drivers of differential proximity to wildfire zones, such as the broader socioeconomic inequities or policies that drove residential location and segregation. The subgroup enumeration presented in this study is specific to California, is not nationally representative, and should be interpreted as such. For example, the “other” Melanesian, Micronesian and Polynesian categories represent heterogeneous populations whose composition is specific to California and not nationally representative. Lastly, consistent with the 2010 Census, which collected Hispanic or Latino origin as ethnicity rather than race [34], this analysis did not include a separate Hispanic or Latino race group; rather, we focused on the OMB definitions of race, where people of any included race group may be of any defined ethnicity (i.e., Hispanic or non-Hispanic). The primary goal of our study was to understand climate exposures among disaggregated subgroups of AAs and NHPIs, which reveals heterogeneity that is often concealed in aggregated analyses. Given this goal, we decided to use only the race variable from the Census and not the Hispanic ethnicity variable. However, examination of exposures among populations with Hispanic/Latino versus non-Hispanic/non-Latino ethnicity is an important area for future research, especially disaggregating Hispanic/Latino ethnicity to examine disparities by various countries of origin. While this is beyond the scope of the present paper, it represents a potential opportunity for future research.

Our results have implications for California’s and US environmental policy, given the critical role the state plays in adopting policies to mitigate air pollution [56]. Apart from annual exposure to total PM_2.5_ exceeding EPA’s newly enacted recommended maximum safe level of 9 µg/m³ across all race groups, we identified substantial disparities within disaggregated groups. We also found race disparities for wildfire-specific PM_2.5_ among disaggregated AA and NHPI groups that showed different patterns than for total PM_2.5._ To achieve the EPA’s aim to provide all people with the same level of protection against environmental hazards [57], future policies should not only improve overall air quality but also prioritize the most affected communities [58,59]. Spatial patterns of environmental burden are highly persistent across time. Longitudinal national studies show that air pollution disparities persist despite overall declines in pollution, indicating structurally durable exposure inequities [60,61]. Communities historically burdened by pollution tend to remain disproportionately exposed due to stable drivers such as land use and infrastructure siting. Critically, prior persistence analyses rely on aggregated racial/ethnic categories and do not examine disaggregated Asian or Pacific Islander populations. Our study addresses this gap by providing the first detailed assessment of long-standing exposure inequities within these subgroups. From a policy standpoint, our findings suggest that efforts to advance environmental justice through policy and community-based actions should include the most impacted communities at the decision-making table. Importantly, these efforts should not treat AAs and NHPIs as monolithic, rather, resources and attention need to be directed toward those communities most disproportionately affected by PM_2.5_ exposure, including Hmong, Fijian, Laotian, Bhutanese, and Marshallese populations. The principle of environmental and restorative justice demands that those communities most at risk for environmental harms be meaningfully involved and have central roles in decision-making to address those harms [58]. Disaggregating AAs and NHPIs reveals which specific communities need protection and inclusion in efforts to achieve environmental justice. Furthermore, our estimates derived from decennial Census data utilizing the most complete racial subgroup enumeration available, allowing us to identify otherwise hidden communities. California's AB 1726 already requires the disaggregation of health data for many of the small subgroups reported here, including Hmong, Bangladeshi, Fijian, and Tongan populations [62]. This reinforces the broader movement toward data disaggregation as a tool for revealing hidden disparities and guiding community response [63].

These efforts should further consider that increased wildfires due to climate change will impact some communities more. The historical subgroup-specific exposure information presented here enables agencies and community-serving organizations to target funding and interventions to the communities carrying long term exposure burden and to mobilize community assets to address shared vulnerabilities [22,33,56]. Future community-based research should aim to better capture community members’ specific exposure risks and risk perceptions to inform equitable policy and programmatic solutions.

## 5 Conclusion

In conclusion, our study establishes a historical baseline of total and wildfire-specific PM_2.5_ exposure disparities among disaggregated AA and NHPI groups in California during the study period. The results highlight the necessity of disaggregated race and ethnicity data to accurately characterize environmental inequities and to identify communities that have experienced disproportionate burdens. The wildfire-driven exposure burden continued to grow since our study period, underscoring the need for ongoing, disaggregated exposure surveillance at local, state, and national levels.

## Funding statement

This research was funded by the National Cancer Institute of the National Institutes of Health grant number 3P20CA253254-03S2 (PI: Sora Park Tanjasiri) and the UC Irvine Institute for Clinical & Translational Science Pilot Study Award (PI: Brittany Morey). The views expressed in this paper do not necessarily reflect those of the funders.

## Data sharing statement

Data described in the manuscript, code book, and analytical code will be made available upon reasonable request to the authors.

## CRediT authorship contribution statement

**Minhazul Mohsin:** Writing – review & editing, Writing – original draft, Visualization, Software, Methodology, Formal analysis, Conceptualization. **Mengyi Li:** Writing – review & editing, Visualization, Software, Methodology, Investigation, Formal analysis, Data curation. **Jun Wu:** Writing – review & editing, Validation, Supervision, Resources, Project administration, Methodology, Data curation, Conceptualization. **Sora Park Tanjasiri:** Writing – review & editing, Supervision, Resources, Project administration, Funding acquisition, Conceptualization. **Brittany N. Morey:** Writing – review & editing, Validation, Supervision, Resources, Project administration, Methodology, Investigation, Funding acquisition, Data curation, Conceptualization.

## Declaration of competing interest

The authors declare that they have no known competing financial interests or personal relationships that could have appeared to influence the work reported in this paper.
